# An Integrated Transcriptomics and Lipidomics Analysis Reveals That Ergosterol Is Required for Host Defense Against Bacterial Infection in *Drosophila*

**DOI:** 10.3389/fimmu.2022.933137

**Published:** 2022-07-07

**Authors:** Zihao Deng, Yanyang Yang, Jiazhen Luo, Biling Zhang, Jiyong Liu, Guanghou Shui, Renjie Jiao, Chuanxian Wei

**Affiliations:** ^1^ Sino-French Hoffmann Institute, School of Basic Medical Sciences, Guangzhou Medical University, Guangzhou, China; ^2^ State Key Laboratory of Molecular Developmental Biology, Institute of Genetics and Developmental Biology, Chinese Academy of Sciences, Beijing, China; ^3^ The State Key Laboratory of Respiratory Disease, Guangzhou Medical University, Guangzhou, China

**Keywords:** *Drosophila*, innate immunity, lipid metabolism, lipidomics, transcriptomics, ergosterol, ACSL

## Abstract

Animals adjust their lipid metabolism states in response to pathogens infection. However, the underlying molecular mechanisms for how lipid metabolism responds to infection remain to be elusive. In this study, we assessed the temporal changes of lipid metabolism profiles during infection by an integrated transcriptomics and lipidomics analysis. Ergosterol is identified to be required for proper host defense to pathogens. Notably, ergosterol level is increased in the hemolymph upon bacterial infection. We show that the increase of ergosterol level by food supplement or genetic depletion of Acsl, a long-chain fatty acid-CoA synthetase, promotes host survival against bacterial challenges. Together, our results suggest a critical role of lipid metabolism adaption in the process of host defense against invading pathogens.

## Introduction

Animals evolve to form a set of sophisticated defense systems to cope with a variety of environmental stresses, such as pathogens infection. Innate immunity is the first line and most ancient host defense system against invading pathogens, including bacteria, fungi and viruses (1). The striking conservation of genetic regulation between flies and mammals together with the well-established genetic resources have made *Drosophila* an attractive model organism to decipher the principles of innate immune response (2, 3). In *Drosophila*, immune response comprises cellular and humoral immunity. In cellular immunity, hemocytes phagocytose or encapsulate, and trigger melanization, to destroy the invading pathogens (4). Humoral immunity, on the other hand, is responsible for the production of antimicrobial peptides (AMPs) mainly from the hemocytes and fat body, through the classical Toll and Imd signaling pathways (5). Although remarkable progress has been achieved during the past three decades in elucidating the underlying mechanisms for innate immune response and regulations, how the immune response activity is regulated physiologically remains largely unknown.

Innate immune system is critical for host survival, yet energetically expensive for a full protection during immune challenges, requiring proper re-distribution of energy (6). Upon pathogen invasion, *Drosophila* hemocytes sense and initiate a metabolic switch to aerobic glycolysis for boosting the immune response (7, 8). Furthermore, the activation of the Imd pathway and/or the Toll pathway in fat body disrupts insulin signaling, results in decreased triglyceride storage and impaired animal growth (9–11). More recently, dSTING, the conserved antiviral signaling pathway in both flies and humans (12, 13), is also reported to regulate lipid metabolism by modulating fatty acid synthesis (14, 15). The infection-induced metabolic adaption and reallocation of energy is indispensable for immune responses, because blocking the metabolic adjustment has been reported to impair host defense against pathogens (16).

Emerging evidence has established a linkage between lipid metabolism and innate immune response, however, the molecular mechanisms by which infection leads to lipid metabolism adaption and how lipid metabolites are involved in immune response are not well understood. In order to systemically assess the molecular connection between these two indispensable processes, here, we performed a time-course study for lipid metabolism genes and metabolites profile dynamics during bacterial infection by an integrated transcriptomics and lipidomics analysis, and identified ergosterol as a novel lipid metabolite involved in proper host defense to bacterial infection.

## Materials and Methods

### Fly Strains and Husbandry

The following fly stocks were used: The wild-type *w^1118^* (#3605) flies were obtained from Bloomington *Drosophila* Stock Center. Acsl^THU2816^ and Acsl^3222^ RNAi lines were obtained from TsingHua Fly Center (THFC) and Vienna *Drosophila* Resource Center (VDRC). The *key^1^*, *MyD88^c03881^* and *w; pUbi-Gal4; pTub-Gal80^ts^* lines were obtained from Dominique Ferrandon’s lab in Institut de Biologie Moléculaire et Cellulaire (IBMC). Unless specifically noted, all flies were kept on standard cornmeal food with yeast (refer to BDSC Cornmeal Food recipe) at 25°C. For yeast-free (YF) food, yeasts were not added to the standard cornmeal food. For ergosterol food, ergosterol (Macklin) was added to the yeast-free cornmeal food with ergosterol (100 mM). For bacterial infection, the newly eclosed flies (within 2 hour) were collected and transferred to YF, YF+ergosterol and normal food (NF), respectively and kept at 25°C for 3 days before bacterial infection. For Acsl RNAi experiment, Ubi-Gal4, UAS-Acsl RNAi, Tub-Gal80^ts^ and control flies were kept at 29°C for 5 days before bacterial infection.

### Bacterial Strains

Gram-negative bacteria *Erwinia carotovora carotovora-15* (*Ecc15*, renamed as *P. carotovorum*) strain was obtained from Lei Pan’s lab in Institut Pasteur of Shanghai, Chinese Academy of Sciences (17). *Serratia marcescens* (*S.m*) (# 1.2818) and *Enterococcus faecalis* (*E.fa*) (#1.2135) were obtained from the China General Microbiological Culture Collection Centre (CGMCC).

### Bacterial Infection

*Ecc15*, *S.m* and *E.fa* were cultured in standard LB medium overnight, with *Ecc15* and *S.m* incubated at 30°C and *E.fa* at 37°C. Before infection, bacteria were washed by 1xPBS for 3 times and diluted to the indicated optical density (OD_600_) of 50, 2 and 0.1, respectively. For infection experiments, flies were anesthetized on CO_2_ pad before bacteria was injected quantitatively into the fly thorax using a microinjector (Nanoject III, Drummond Scientific Company). The injection volume of *Ecc15*, *S.m* and *E.fa* were 15, 2.3 and 9.2 nL per fly, respectively. Of note, the injection volume of *Ecc15* in **Figure 4A** was 23 nL per fly.

### Bacterial Load

To determine the CFU of *Ecc15*, *S.m* and *E.fa-*infected flies, individual fly was first homogenized gently in 200 μL 1xPBS at the indicated time points. In brief, a sterile zirconia bead (φ=3.0 mm) was added into the sample with 200 μL pre-cooled 1xPBS, then the sample was crashed by vibrating the zirconia bead with frequency of 30Hz for 30s in a Retsch MM400 grinding mixer. Then, 10 μL of homogenates were serial diluted at 1:10^4^. 10 μL of the diluted-homogenates were placed on standard LB agar plate at 37°C overnight before counting. Each diluted sample was performed in duplicate.

### qPCR

To measure mRNA levels of indicated fly genes, ~7 flies were collected for homogenate preparation. For homogenate preparation, a sterile zirconia bead was added into the sample with 200 μL pre-cooled RNAiso, then the sample was crashed by vibrating the zirconia bead with frequency of 30Hz for 3 min in a Retsch MM400 grinding mixer. Homogenates of fly for each treatment were then extracted by RNAiso Plus kit (TAKARA). RNA (1 μg) was reverse transcribed using PrimeScript™ RT reagent kit (TAKARA). qPCR analyses were preformed using the TB Green premix Ex Taq™ II kit on a BIO-RAD C1000 Touch™ Thermal Cycle. And the expression levels of target genes were normalize to *rp49* (data are presented as ΔCT, 2^(Ct^rp49^ - Ct^target genes^)). Primers used were in **Supplementary Table 2**.

### Hemolymph Extraction

The *Ecc15*-infected *w^1118^* female flies (OD=50, 15 nL) and the corresponding PBS-injected (control) flies were poked by needle at thorax and then placed at a 500 μL Eppendorf tube with a hole in the bottom, which was inserted in a 1.5 mL tube. Flies were centrifuged at 5,500 rpm for 15 min. The yielded hemolymph was gently mixed with TBST (0.1% Tween-20) and stored at -80°C until use.

### RNA-Seq and Bioinformatics Analyses

The total RNA of *Ecc15*-infected and PBS-injection control *w^1118^* female flies were extracted at 12, 24, 48 and 72 hpi. The eukaryotic mRNA was enriched by Oligo (dT) beads, and rRNA was removed by Ribo-Zero™ Magnetic Kit (Epicentre Madison, WI, USA). Then, the RNA was quantified, reverse transcribed and sequenced by Illumina sequencing platform. These eukaryotic mRNA enrichment, rRNA elimination, cDNA library preparation and sequencing procedures were performed by Guangzhou Genedenovo Biotechnology Co., Ltd. The raw sequencing data were filtered by trim galore software (v0.6.4) to remove plausible remaining adapter sequences in reads and low quality (Q-value<=20) reads. The resulting clean data were aligned to fly’s genome dm6 using the STAR software (v2.7.2b) (18). Gene expression levels (gene counts and TPM- Transcripts Per Million) were quantified by rsem software (v1.3.1) (19). The following bioinformatics analyses were performed base on R software (v3.5.3). In detail, Weighted Correlation Network Analysis (WGCNA) was performed based on WGCNA package (v1.69) (20). In brief, the TPM gene expression matrix generated by rsem software was log2 transformed and was transposed; Second, soft thresholding powers were then calculated by WGCNA::pickSoftThreshold function; Third, automatic network construction and module detection were calculated by WGCNA::blockwiseModules function with soft thresholding powers calculated above (in this study, sft=14); Forth, the correlation value between module and time-course treatment (such as *Ecc15* 12 h, PBS 12 h et al.) were calculate by WGCNA::cor function; In the last step, the visualization of the correlation ship modules and time-course treatment by WGCNA:: labeledHeatmap function. Gene Ontology (GO) and Gene Set Enrichment Analysis (GSEA) analysis were performed using clusterProfiler package(v3.10.1) (21). In brief, for GO analysis, the indicated gene list were imported to the clusterProfiler::enrichGO function to preform biological process GO terms enrichment analysis. For GSEA analysis, the down-regulated genes of PBS *vs Ecc15* 12 h/24 h were imported to clusterProfiler::gseGO function to perform GSEA analysis. The visualizations of lipid metabolic process (GO:0006629) were plotted by clusterProfiler::gseaplot function. Transcription factor binding motifs enrichment analysis was performed by RcisTarget package (v1.2.1) (22). In brief, the lipid metabolism process related-genes identified by WGCNA analysis in the red module were imported to the RcisTarget::cisTarger function to identify DNA motifs that were significantly over-represented in the gene-set. In this step, the Hnf4 binding motif was identified (NES=9.72 and AUC =0.249) using dm6_motifRanking_mc8nr (https://resources-mirror.aertslab.org/cistarget); Second, to get the incident matrix of significant genes which were highly ranked for Hnf4 binding motif, RcisTarget::getSignificantGenes was used. In the last, the visualization of the incident matrix was plotted by Cytoscape software (v3.7.2)

Differential expression genes (DEGs) were identified by DEseq2 package (v1.22.2) (23). In brief, the gene counts expression matrix generated by rsem software were imported to the DESeq2::DESeq function to calculate differential expression analysis based on the Negative Binomial distribution, then the gene expression comparison results of each group (such as such as “PBS *vs Ecc15* 12 h”, “PBS *vs Ecc15* 24 h” et al.) were obtained by DESeq2::result function.

### Lipid Extraction

Lipid were extracted from *Drosophlia* hemolymph using a modified version of the Bligh and Dyer’s method as described previously (24). In brief, add 750 µL of chloroform: methanol 1:2 (v/v) with 10% deionized water to samples. Then samples were incubated at 1,500 rpm for 1 h at 4°C. After the incubation, 350 µL of deionized water and 250 µL of chloroform were added to samples to induce phase separation. The samples were then centrifuged and the lower organic phase containing lipids was extracted and transferred into a clean tube. Lipid extraction was repeated once by adding 500 µL of chloroform to the remaining aqueous phase, and the lipid extracts were pooled into a single tube and dried in the SpeedVac under OH mode. Samples were stored at -80°C until further analysis.

### Lipidomic Analyses

Polar lipids were analyzed using an Exion UPLC system coupled with a triple quadrupole/ion trap mass spectrometer (6500 Plus Qtrap; SCIEX) as described previously (25–27). Separation of individual lipid classes of polar lipids by normal phase (NP)-HPLC was conducted using a Phenomenex Luna 3µm-silica column (internal diameter 150 × 2.0 mm) with the following conditions: mobile phase A (chloroform: methanol: ammonium hydroxide, 89.5:10:0.5) and mobile phase B (chloroform: methanol: ammonium hydroxide: water, 55:39:0.5:5.5). MRM transitions were set up for comparative analysis of various polar lipids. Individual lipid species were quantified by referencing to spiked internal standards. PC-d31(16:0/18:1), PE-d31(16:0/18:1), PS-d31(16:0/18:1), PI-d31(16:0/18:1), PA(17:0/17:0), PG-d31(16:0/18:1), C17-Cer, C12-PECer were obtained from Avanti Polar Lipids. Dioctanoyl phosphatidylinositol **(PI)** (16:0-PI) was obtained from Echelon Biosciences, Inc. Glycerol lipids including diacylglycerols (DAGs) and triacylglycerols (TAGs) were quantified using a modified version of reverse phase HPLC/MRM. Separation of neutral lipids were achieved on a Phenomenex Kinetex-C18 2.6 µm column (i.d. 4.6x100 mm) using an isocratic mobile phase containing chloroform: methanol: 0.1 M ammonium acetate 100:100:4 (v/v/v) at a flow rate of 170 µL for 17 min. Levels of short-, medium-, and long-chain TAGs were calculated by referencing to spiked internal standards of TAG (16:0)3-d5 and TAG (18:0)3-d5 obtained from CDN isotopes, respectively. DAGs were quantified using d5-DAG16:0/16:0 as internal standards (Avanti Polar Lipids). Free cholesterols and cholesteryl esters were analyzed as described previously with d6-cholesterol and d6-cholesteryl ester (CE) (CDN isotopes) as internal standards.

## Results

### Time-Course Lipidomic Profiling of *Drosophila* Hemolymph After *Ecc15* Infection Reveals a Dynamic Remodeling of Lipid Profiles

Immune challenges induce systemic adaptive changes of lipid metabolism to coordinate the proper mobilization of energy for host defense. *Drosophila* hemolymph, the open and circulating system, is the major platform for materials and energy exchange between tissues/organs (28). Thus, lipid metabolites profiling of the hemolymph during bacterial infection will provide important hints to assess the lipid metabolic status at the organismal level.

**Figure 1:**
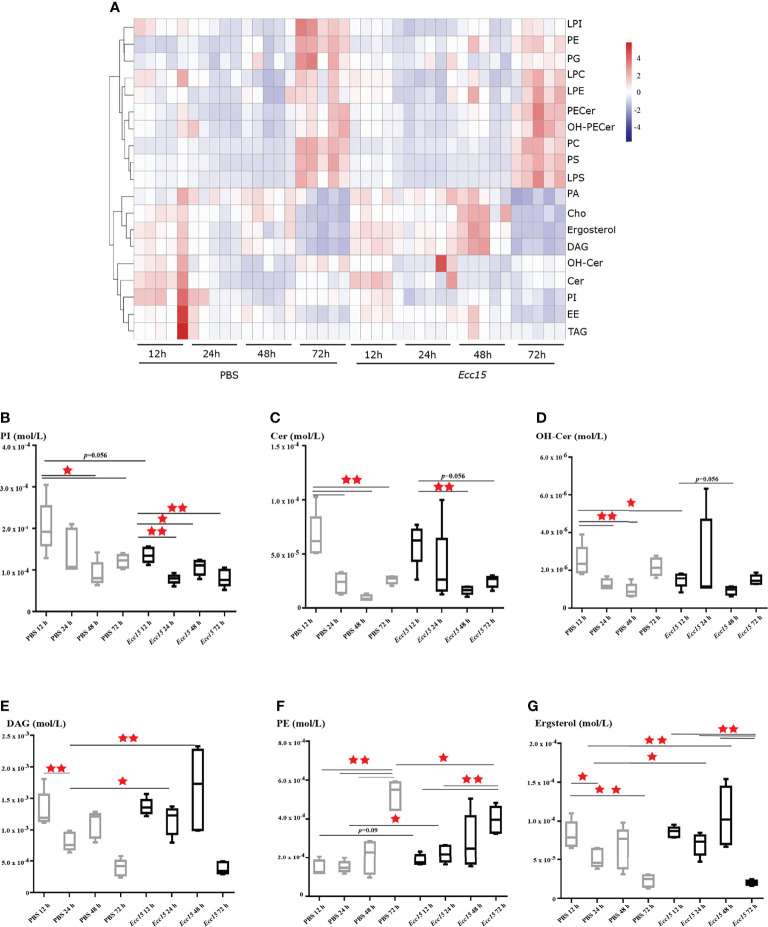
Time-course lipidomic profiling of *Drosophila* hemolymph after *Ecc15* infection reveals a dynamic remodeling of lipid profiles. **(A)**. Heatmap for selected lipid species profiles at indicated time points (12, 24, 48, 72 hpi) in control (PBS) and *Ecc15*-challenged (*Ecc15*) flies. 5 duplicates were performed for each group. **(B–G)**. Box-plot of phosphatidylinositols (PI) **(B)**, Ceramides (Cer) **(C)**, Hydroxyceramide (OH-Cer) **(D)**, Diacylglycerols (DAG) **(E)**, Phosphatidylethanolamines (PE) **(F)**, ergosterol **(G)** at indicated time points (12, 24, 48, 72 hpi) in control (PBS) and *Ecc15*-challenged (*Ecc15*) flies. *, p<0.05; **, p<0.001. (wilcox test).

To this end, we collected the hemolymph from *Ecc15* (renamed as *P. carotovorum*)-challenged and control (PBS) flies at different time points (12, 24, 48, 72 hpi), and performed a quantitative lipidomic analysis to assess their dynamic lipid metabolites profiles, including glycerides, phospholipids, glycerophosphatides, sphingolipids and sterols. Our lipidomic analysis revealed that several lipids, mainly phospholipids, sphingolipids and sterols, were significantly changed when flies were challenged with *Ecc15* infection, such as diacylglycerols (DAGs), ceramides (Cer), hydroxyceramide (OH-Cer), phosphoinositides (PIs), and phosphatidylethanolamines (PEs). These results indicate that a systemic adaptive change of lipid metabolites occurs in *Ecc15*-challenged flies (**Figure 1A**, **Supplementary Table 1**). In particular, the level of PIs, commonly as membrane ingredients and intracellular signal transducing molecules, was down-regulated in both clean-wounded and *Ecc15*-infected flies (**Figure 1B**). The facts that supplementation of the diet with PIs, not other lipids, suppresses the proinflammatory cytokine levels in mammals (29), imply that the decrease of PIs in early stage of challenges is an active response of host to release the brakes for a full activation of immune response. Additionally, Cer and OH-Cer showed similar trend with PIs after infection (**Figures 1C, D**), which suggests that flies may increase the threshold to the oxidative stress damages through reducing the Cer and OH-Cer levels, as ceramides act as mediators of oxidative stress and inflammation in several human diseases (30–32). Infection-induced DAGs loss in the hemolymph (**Figure 1E**), likely either through renal purging of DAGs to prevent from oxidative stresses (33) or enhanced intake by surrounding tissues for energy supply (34).

Unlike PIs and Cer, the level of PEs, the second most abundant glycerophospholipid in flies, was significantly increased in *Ecc15*-infected flies compared to control (**Figure 1F**). Interestingly, we found that the *Ecc15*-infected flies contained high level of ergosterol, the major sterol in flies, in the hemolymph, compare with PBS-injected flies (**Figure 1G**).

Taken together, our temporal lipidomic analysis reveals that activation of immune response by bacterial stimuli results in the intense dynamic remodeling of lipid metabolism profiles, leading to the reallocation of large quantities of lipid metabolites during host defense.

### Ergosterol Is Required for Host Defense of Bacterial Infection in *Drosophila*

**Figure 2:**
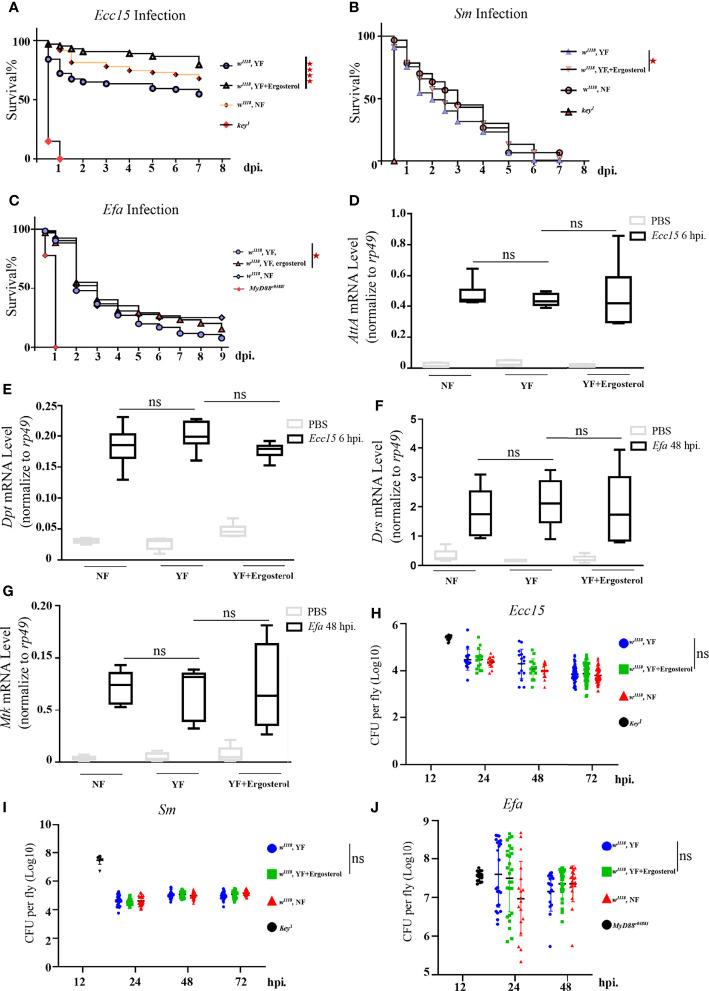
Ergosterol is required for host defense of bacterial infection in *Drosophila*. **(A–C)**. Survival curves of *w^1118^* and *key^1^* or *Myd88^c03881^* flies infected with *Ecc15* **(A)**, *S.m* **(B)**, and *E.fa* **(C)**, reared on different food. ****, p<0.0001 (Log-rank test). **(D, E)**. Q-PCR plot of *AttA* and *Dpt* mRNA level in flies infected with *Ecc15* at 6hpi, reared on different food. ns, not significant. (One-way ANOVA test with a Sidak test). **(F, G)**. Q-PCR plot of *Drs* and *Mtk* mRNA level in flies infected with *E.fa* at 48hpi, reared on different food. ns, not significant. (One-way ANOVA test with a Sidak test). **(H–J)**. CFU assay of *w^1118^* and *key^1^* or *Myd88^c03881^* flies infected with *Ecc15* **(H)**, *S.m* **(I)**, and *E.fa* **(J)**, reared on different food. ns, not significant. (One-way ANOVA test with a Sidak test). NF, normal food; YF, yeast-free food; YF+ergosterol, yeast-free food containing ergosterol (100 mM).

Above results indicate that ergosterol level in hemolymph is significantly increased during *Ecc15* infection, implying a potential role of ergosterol in host defense to bacterial infection. Unlike the well-characterized ergosterol synthesis pathway in fungi, there are no genes encoding the enzymes required for ergosterol *de novo* synthesis in *Drosophila* genome (35). Thus, ergosterol can only be ingested by flies from food. In standard fly food, yeast is the main source for ergosterol (35). Therefore, to investigate the potential role(s) of ergosterol in immune response, we reared the adult flies on yeast-free (YF) food and YF food with 100 mM ergosterol supplement (YF + ergosterol), respectively, and examined their susceptibility to *Ecc15* infection. We found that flies reared on ergosterol food were more resistant to *Ecc15* infection, as indicated by the extended survival curve (**Figure 2A**). Moreover, ergosterol supplement also rendered flies more resistant to *S.m* and *E.fa* infection (**Figures 2B, C**). Further, we wonder whether the increased host defense to infection was contributed by their enhanced immune response activity by ergosterol supplement. To address this question, we determined the transcriptional level of antimicrobial peptides Attactin A (*AttA*) and Diptericin (*Dpt*), which are indicators for the activation of Imd immune response pathway after *Ecc15* infection. However, ergosterol feeding did not increase the *AttA* and *Dpt* mRNA level (**Figures 2D, E**). Besides, ergosterol feeding did not change the bacterial load (**Figure 2H**). Similar results were observed in *S.m* and *E.fa*-challenged flies (**Figures 2F, G, I, J**). Together, these results demonstrate that ergosterol, as a potent immune modulator, enhances host defense against bacterial infection, without changing the immune response strength.

### Time-Course Transcriptomic Analysis Reveals That Changes of Lipid Metabolism Related Gene Expression Profiles Occur in Response to *Ecc15* Infection

Next, we wondered how the ergosterol level in the hemolymph was regulated during infection. It is reported that bacterial infection decreases the feeding behavior by triggering food avoidance in *Drosophila* larvae (36) and pathogens infection also initiates a rapid renal purging of hemolymph lipids to reduce the lipid peroxidation level, lessening tissue damages (33). These studies suggest that the elevated ergosterol level in the hemolymph after infection is unlikely due to increased feeding or decreased defecating behaviors in response to pathogens invasion, raising the possibility that infection induced ergosterol level change is regulated intrinsically by host lipid metabolism machineries.

**Figure 3:**
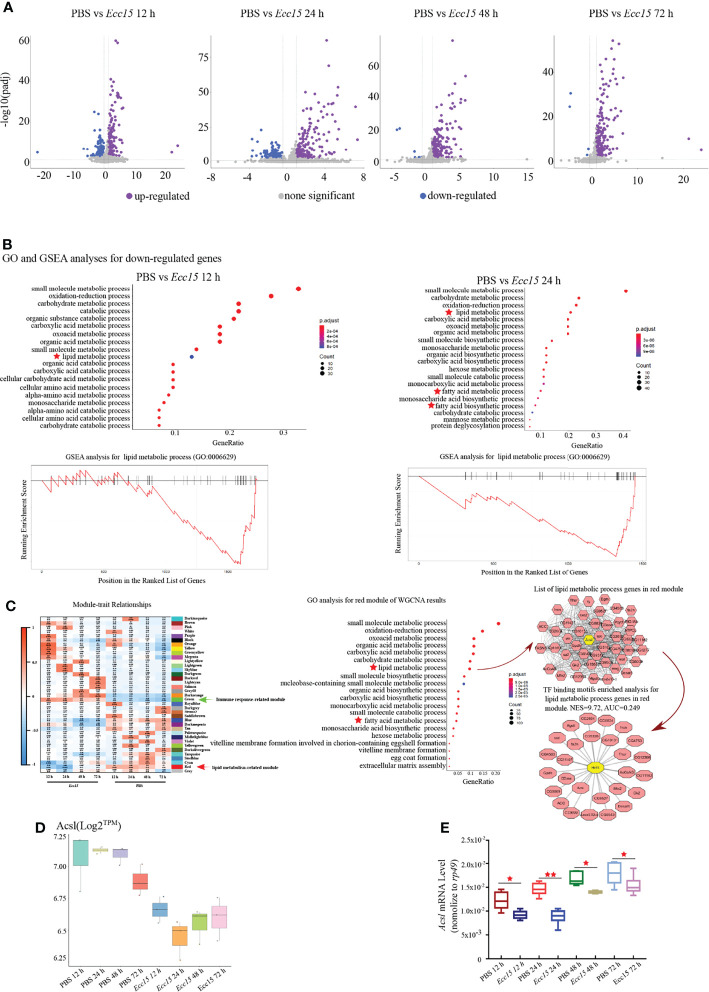
Time-course transcriptomic analysis reveals that changes of lipid metabolism related gene expression profiles occur in response to *Ecc15* infection. **(A)**. Volcano plot of differentially expressed genes (DEGs) with >2-fold up- or >0.7-fold down-regulation changes (p-adj<0.001) in flies infected with *Ecc15* at indicated time points (12, 24, 48, 72 hpi), compared with control flies. **(B)**. GO analysis (upper panel) for the down-regulated genes in **(A)** and GSEA analysis (lower panel) for genes with GO termed “lipid metabolic process” (GO:0006629) in down-regulated genes at indicated time points (12, 24 hpi). **(C)**. Left panel, WGCNA analysis for time-course transcriptomic data. Middle panel, GO analysis for Module Red of WGCNA result. Right panel, hub-gene analysis (right-upper) and TF binding motifs enrichment analysis (right-lower) for genes with GO termed “lipid metabolic process” (GO:0006629) in Module Red. **(D, E)**. Transcripts per million (TPM) **(D)** and Q-PCR **(E)** plot of *Acsl* mRNA level in control (PBS) and *Ecc15*-challenged (*Ecc15*) flies at indicated time points (12, 24, 48, 72 hpi). *, p < 0.05; **, p < 0.001. (One-way ANOVA test with a Sidak test).

To investigate the regulatory mechanism for ergosterol level, we collected the whole flies at indicated time points (12, 24, 48 and 72 hpi) after *Ecc15* infection, and performed a temporal transcriptomic analysis for their global RNA expression profiles (**Supplementary Table 3**). Large number of genes were upregulated after *Ecc15* infection, compared with PBS-injected control flies (**Figure 3A**). Interestingly, along with the infection time, the numbers of upregulated genes increased (154, 170, 203 and 217) while the numbers of down-regulated decreased (146, 127, 6 and 4) (**Figure 3A**), indicating the different response profiles at different time after infection.

Next, we performed a Gene Ontology (GO) analysis for the up-regulated genes in *Ecc15*-challenged flies. The GO analysis revealed that genes related to host defense were enriched (**Supplementary Figures S1A, B**), as expected. Notably, genes involving lipid metabolism was significantly enriched in the downregulated genes (designated as “lipid metabolic process”, “fatty acid metabolic process” and “fatty acid biosynthetic process”) (**Figure 3B**; **Supplementary Table 4**), suggesting that lipid metabolism was extensively regulated during infection. Next, we performed Gene Set Enrichment Analysis (GSEA) analysis for the down-regulated genes after *Ecc15* infection, genes involving lipid metabolic process were highly enriched (**Figure 3B**). These data indicated a systemic adaption in host lipid metabolism during bacterial infection.

To further identify the genes or gene sets whose expression profiles were most tightly correlated with host defense in the bacterial infection processes, a weighted correlation network analysis (WGCNA) was performed. We identified 37 signature modules with distinctive correlation features to *Ecc15* infection (**Figure 3C**). Module Green was significantly positively correlated to infection at 12 and 24 hpi, in which the immune related genes (49 genes in GO:0006955) were significantly enriched, suggesting that flies initiated an extensive immune response against bacterial stress at a relatively early stage (**Figure 3C**, **Supplementary Figure 2** and **Supplementary Table 5**). Interestingly, Module Red, which exhibited significantly negative correlation with *Ecc15* infection, was identified (**Figure 3C**). In this module, a bunch of lipid metabolism-related genes (47 genes in GO:0006629, designated as “lipid metabolic process” and “fatty acid metabolic process”) was enriched (**Figure 3C** and **Supplementary Table 6**). Further network analysis identified Acsl, a long-chain fatty acid-CoA synthetase, as a hub gene in the Module Red. Both RNA-Seq and qPCR results confirmed that Acsl were down-regulated in *Ecc15*-infected flies (**Figures 3D, E**).

To find out the key regulator(s) responsible to these down-regulated lipid metabolic genes in this module, we performed a transcription factor (TF) binding motifs enrichment analysis, and the result supported a pivotal role of HNF4, a master regulator for lipid mobilization and fatty acid beta-oxidation, as a core transcription factor for lipid metabolism change during *Ecc15* infection (**Figure 3C**). Altogether, these data demonstrate that host coordinates multiple biological machineries, especially lipid metabolism processes, to efficiently fight with pathogen invasion.

### Flies With *Acsl* Depletion Are More Resistant to Bacterial Infection Than the Wild Type

**Figure 4:**
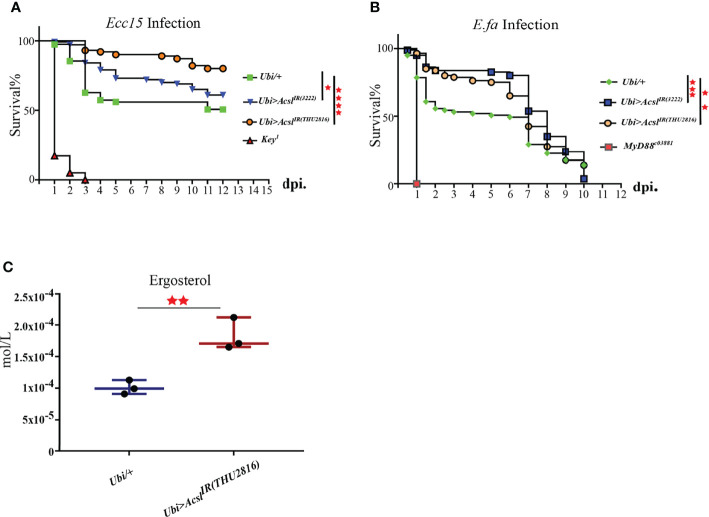
Flies with *Acsl* depletion are more resistant to bacterial infection than the wild type. **(A)**. Survival curves of Acsl RNAi and *key^1^* flies infected with *Ecc15*. *, p<0.05; ****, p<0.001. (Gehan-Breslow-Wilcoxon test). **(B)**. Survival curves of Acsl RNAi and *Myd88^c03881^* flies infected with *E.fa*. **, p<0.01; ***, p<0.005. (Gehan-Breslow-Wilcoxon test). **(C)**. Box plot of ergosterol in control and Acsl RNAi flies. **, p<0.01 (wilcox test).

Our previous results have established the pro-survival role of ergosterol in flies challenged with bacteria. Thus, we wonder whether Acsl, the hub gene in infection-induced lipid metabolism profile, also regulates ergosterol level and plays a role in host defense by regulating ergosterol level. Systemic knockdown of Acsl lead to flies lethal, thus, we utilized temperature-sensitive (with Tub-Gal80^ts^) Gal4/UAS system to spare the normal functions of Acsl during development. Taking this system, we ubiquitously knockdown *Acsl* mRNA level in adult flies (**Supplementary Figure 3**), and examined their susceptibility to bacterial infection. Indeed, the survival rates of Acsl RNAi flies were significantly higher than wild type flies against systemic *Ecc15* infection (**Figure 4A**). Notably, Acsl knockdown also rendered flies more resistant when challenged with *E.fa*, a Gram positive bacteria (**Figure 4B**). Next, we collected the fly hemolymph and determined the ergosterol level by LC/MS in Acsl RNAi flies. Ergosterol level in the hemolymph was significantly increased when flies were ubiquitously depletion of Acsl (**Figure 4C**). These results suggest that modulation of ergosterol level through Acsl manipulation regulates the sensitivity of flies to bacterial infection, representing a previously uncharacterized role of Acsl-ergosterol metabolism pathway in host defense to pathogen invasion.

## Discussion

Pathogen invasion triggers an immune response in the host. Flies respond to immune challenges by activating a conserved Toll-Dif or/and Imd-Relish pathway that stimulates synthesis and secretion of substantial antimicrobial peptides (AMPs) into hemolymph (37). Usually, activation of innate immune signaling is energetically demanding, requiring the coordinating of nutritional supply system and host defense system, to achieve efficient pathogens killing. Lipid metabolism has been shown to be involved in providing energy and signal transduction processes during immune responses. In both flies and humans, excessive immune activation leads to metabolic dysregulation, while loss of metabolic homeostasis usually results in the weakening of the immune system, demonstrating an intimate link between lipid metabolism and immune response (38). The coordination of immune and metabolic pathways and functional conservation of these pathways between flies and mammals make *Drosophila* an ideal model for the study of immune-metabolism crosstalk at organismal level.

Metabolic adaption occurs in response to bacterial infection. However, how bacterial infection leads to lipid metabolism changes during infection and what are the main lipid metabolites that involving host defense are still largely unknown. We performed a temporal lipidomic and transcriptomic analysis to investigate the dynamic profiles of lipid metabolic genes and lipid metabolites in the hemolymph of flies challenged with *Ecc15* infection. Systemic metabolic change was observed in *Ecc15*-infected flies, indicating a complicated role of specific lipid metabolites in respond to pathogen invasion.

Lipidomic analysis revealed profound changes of several components of phospholipids, sphingolipids and sterols, such as PIs, PEs, Cer/OH-Cer, DAGs, and ergosterol, implying their potential roles in host defense. Rather than cholesterol, ergosterol is the main sterol in flies (35). We wonder whether the increased ergosterol induced by *Ecc15* infection is functional or not in immune system. Notably, artificially increase of ergosterol level in the hemolymph, by oral feeding or genetic manipulation, significantly enhanced host defense to bacterial infection by promoting host survival. Consistent with our finding, Adrien Franchet et al. found that feeding ergosterol promoted host survival against the microsporidium *Tubulinosema ratisbonensis* through an unknown mechanism (39).

Then, as the main sterol in flies, how does ergosterol function in immune response? We proposed that ergosterol may function as cholesterol substitute in immune response, based on the following: 1) Ergosterol and cholesterol share highly similar molecular structure. 2) They can substitute each other for supporting fly growth and survival. Based on these, it is plausible that ergosterol has the ability to be converted into hormones, such as ecdysone, which plays an important role in promoting innate immune response, by increasing phagocytic activities on blood cells or promoting pattern recognition receptors (i.e. PGRP-LC and PGRP-LE) expression (40). However, regardless of the structural similarity to cholesterol (41), ergosterol cannot be converted into ecdysone, instead, into 24-epi-MaA, dh-methylE and dhMaA, which can also support the whole life cycle of fruit fly (42). Our data showed that ergosterol did not alter AMPs mRNA level and bacterial load, indicating that ergosterol did not promote immune response signaling activation, even though ergosterol or its metabolites showed overlapped functions with ecdysone in supporting animal growth.

Ergosterol is the main sterols in the plasm membrane (PM) in flies, and sterols are required for special microdomain formation, for example, lipid rafts and immune-synapses, on the PM. We wonder whether ergosterol is required for host defense by boosting immune signaling activation through clustering immune components for pathogens recognition and sequential signal transduction. However, our findings are not supportive to this hypothesis due to the observations that ergosterol feeding did not change AMPs expression level. Interestingly, increased ergosterol level caused by *Acsl* depletion in Huang et al.’s study was associated with impaired Dpp signaling (43), implying a context (e.g., tissue, pathway)-dependent action of ergosterol in flies.

Alternatively, ergosterol severs as an integrate component of membrane and promotes membrane fusion (44, 45), raising the possibility that ergosterol tends to participate in maintaining the immune homeostasis and tissue repair (resilience), instead of killing pathogens or activating immune response (resistance). This possibility is supported by the findings that in yeast the ergosterol can sustain membrane integrity by regulating membrane fluidity and permeability (46) through interacting with phospholipids and membrane-anchored proteins (47). Additionally, the tolerance capacity of yeast to environmental stresses is tightly correlated to its intracellular ergosterol levels (48). Further studies are needed to decipher whether ergosterol functions in membrane repair and integrity.

Our further study identified Acsl as the key regulator for lipid metabolism adaption in respond to pathogen infection. It is reported that knockdown of Acsl increased the ergosterol level in fly brains (43). Consistently, our results showed ergosterol level in the hemolymph was increased in Acsl knockdown flies. So, how does Acsl affects ergosterol level *in vivo*? As a key player in fatty acid synthesis with its palmitoyl-CoA ligase activity, Acsl is localized on ER (43, 49), promotes *de novo* formation of lipid droplets (49), which are identified as the central hub integrating and coordinating cellular metabolism and the immune system (50), to cope with intrinsic and extrinsic stresses. Lipid droplets are primarily composed of polar lipids and residual proteins on the monolayer membrane surface with neutral lipids in the core content, including TAG and sterols (51). Interestingly, it is reported that in yeast the ergosterol can be converted into the form of steryl-esters (SEs) and stored in lipid droplets, serving as a sterol pool to maintain the balance of intracellular sterols (52, 53). Although lacking of direct evidence, it is possible that Acsl reduces the free ergosterol level by promoting the ergosterol packing into lipid droplets, while knockdown of Acsl increases ergosterol by breaking down the lipid droplets, releasing ergosterol into the hemolymph. Recently, Huang et al. reported that knockdown of Acsl in the fly brain resulted in significant changes of several lipids, such as ergosterol, phosphoethanolamine ceramide (CerPE), and mannosyl glucosylceramide (MacCer), by an unknown mechanism, impairing signaling transduction (43). In line with our observations, it is suggested that lipid metabolites may work synergistically to deal with intrinsic and extrinsic stresses.

Taken together, our study identified a previously unidentified role of Acsl-ergosterol metabolism axis in immune response, providing a potential alternative way for modulating host defense for infection diseases treatment.

## Data Availability Statement

The datasets presented in this study can be found in online repositories. The names of the repository/repositories and accession number(s) can be found below: Genome Sequence Archive in BIG Data Center (https://bigd.big.ac.cn), Beijing Institute of Genomics (BIG), Chinese Academy of Sciences, under accession number: CRA006720.

## Author Contributions

ZD, RJ and CW conceived the study, designed the experiments, discussed the results, and wrote the manuscript. ZD, YY, JZL, and BZ performed the experiments and analysis. ZD generated the figures. GS provided technical assistance in lipidomic assay. JYL discussed the results. All authors read and approved the manuscript. All authors contributed to the article and approved the submitted version.

## Funding

This study has been financially supported by the National Key R&D Program of China (2021YFA0805800 and 2020YFA0803202 to RJ), the National Natural Science Foundation of China (31970538 to RJ, 32100703 to CW, 32000574), Guangzhou Medical University Discipline Construction Funds (Basic Medicine) (JCXKJS2022A02 to RJ), the 111 Project (D18010 to RJ), the Local Innovative and Research Teams Project of Guangdong Perl River Talents Program (2017BT01S155 to RJ), the Special Innovation Projects of Universities in Guangdong Province (2018KTSCX182 to JL), the Medical Scientific Research Foundation of Guangdong Province (A2019292 to JL), the Natural Science Foundation of Guangdong, China (2017A030310403 to ZD), and the grant of the State Key Laboratory of Respiratory Disease, Guangdong-Hong Kong-Macao Joint Laboratory of Respiratory Infectious Disease (GHMJLRID-Z-202106 to CW).

## Conflict of Interests

The authors declare that the research was conducted in the absence of any commercial or financial relationships that could be construed as a potential conflict of interest.

## Publisher’s Note

All claims expressed in this article are solely those of the authors and do not necessarily represent those of their affiliated organizations, or those of the publisher, the editors and the reviewers. Any product that may be evaluated in this article, or claim that may be made by its manufacturer, is not guaranteed or endorsed by the publisher.

## Supplementary Material

Supplementary Figure 1 GO analysis and Heatmap for up-regulated genes after *Ecc15* infection. **(A1-A4)** GO analysis for the up-regulated genes at indicated time points (12, 24, 48, 72 hpi). **(B)** Heatmap plot for AMPs genes of Imd pathway at indicated time points (12, 24, 48, 72 hpi).

Supplementary Figure 2 GO analysis for genes in Module Green in WGCNA result.

Supplementary Figure 3 Knockdown efficiency of Acsl RNAi lines. Q-PCR plot of *Acsl* mRNA level in control and Acsl RNAi flies. Ubi/w^1118^, *w; pUbi-Gal4/+; pTub-Gal80^ts^/+.* Ubi>Acsl^IR(3222)^, *w; pUbi-Gal4/UAS-Acsl^IR(3222)^; pTub-Gal80^ts^/+*. Ubi> Acsl^IR(THU2816)^, *w; pUbi-Gal4/+; pTub-Gal80^ts^/UAS- Acsl^IR(THU2816)^*. ****, p<0.001. (One-way ANOVA test with a Sidak test).

Supplementary Table 1 List of lipid metabolites detected in lipidomic analysis.

Supplementary Table 2 Primers used in this study.

Supplementary Table 3 Gene expression matrix (TPM and Counts) in *Ecc15* challenged flies at indicated time points.

Supplementary Table 4 Top 50 GO list in down-regulated DEGs gene after *Ecc15* infection.

Supplementary Table 5 Top 50 GO list in WGCNA Green Module.

Supplementary Table 6 Top 50 GO list in WGCNA Red Module.
