# Supplementary figures and images for: An Integrated Transcriptomics and Lipidomics Analysis Reveals That Ergosterol Is Required for Host Defense Against Bacterial Infection in Drosophila
Source: Front Immunol. 2022 Jul 7;13:933137. doi: 10.3389/fimmu.2022.933137 (PMC9301368; doi:10.3389/fimmu.2022.933137)

Fig S1

A1

PBS vs *Ecc15* 12 h

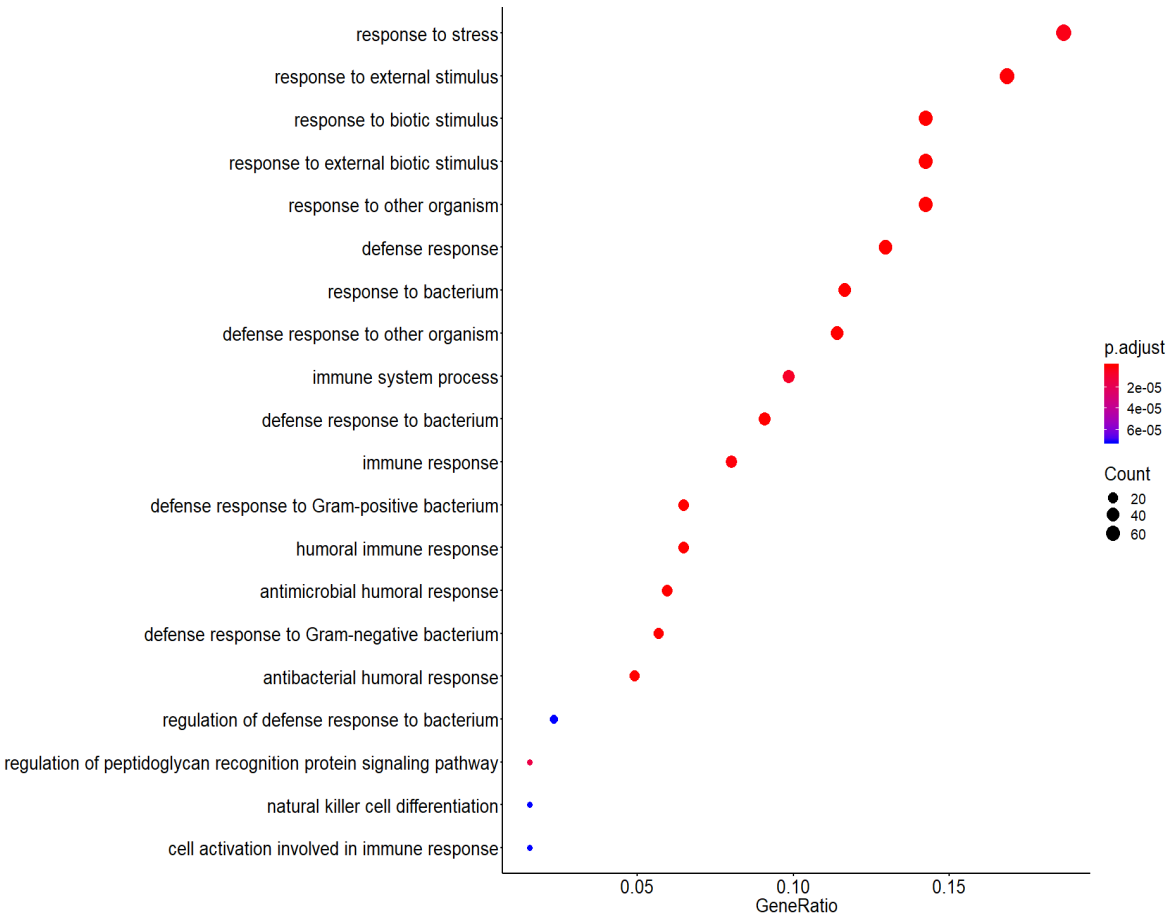

A2

PBS vs *Ecc15* 24 h

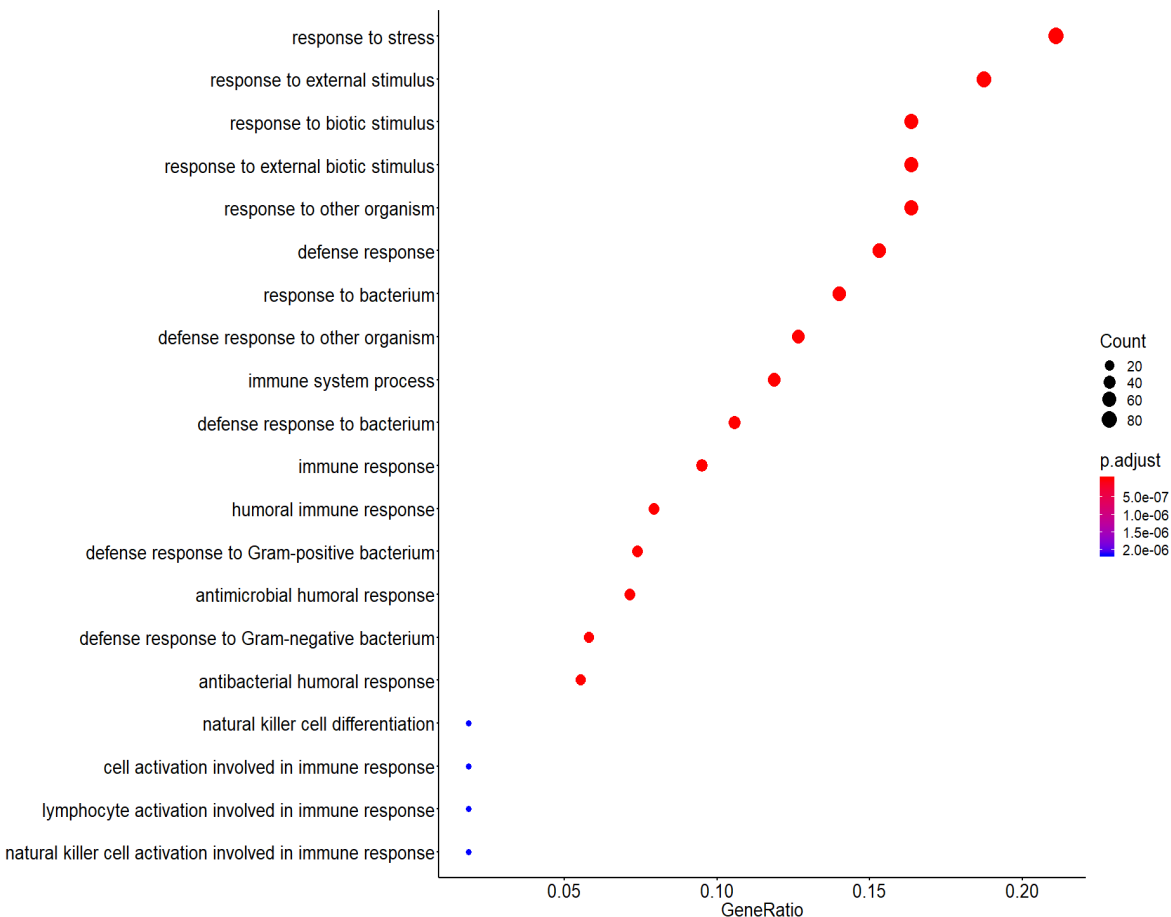

A3

## PBS vs *Ecc15* 48 h

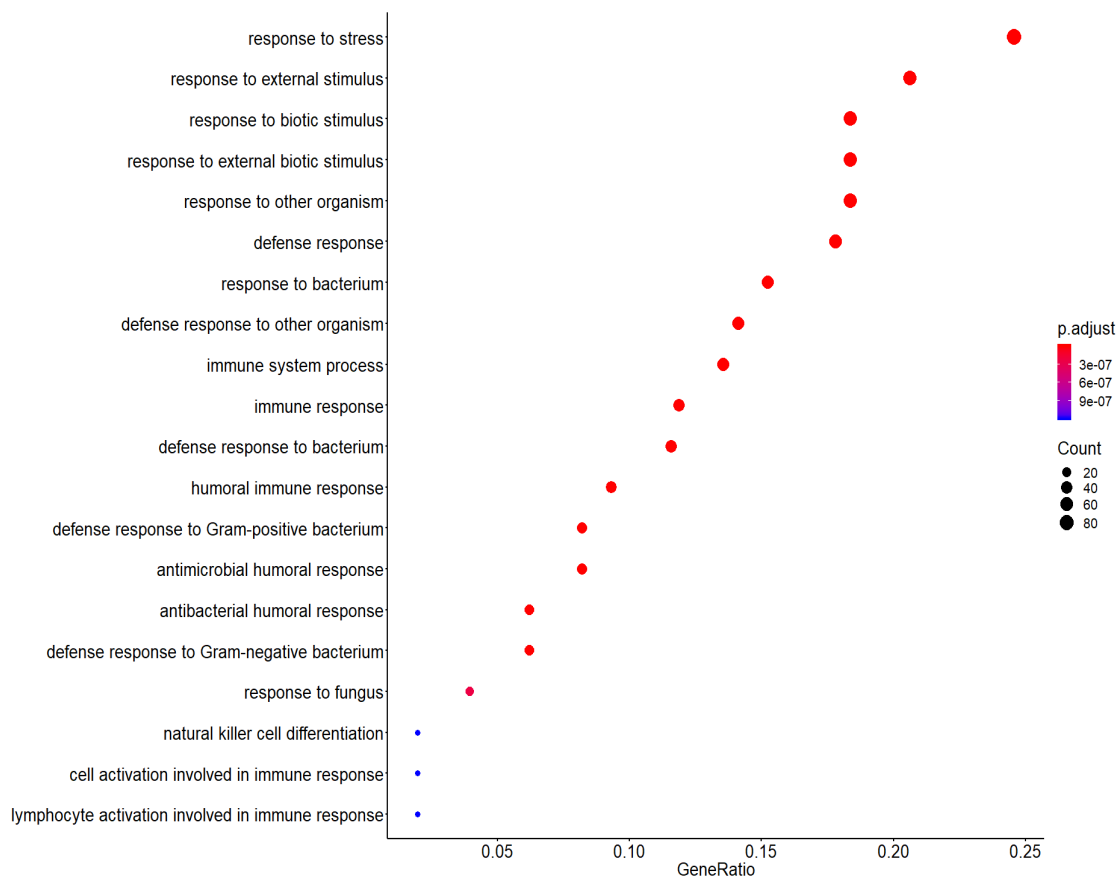

A4

## PBS vs *Ecc15* 72 h

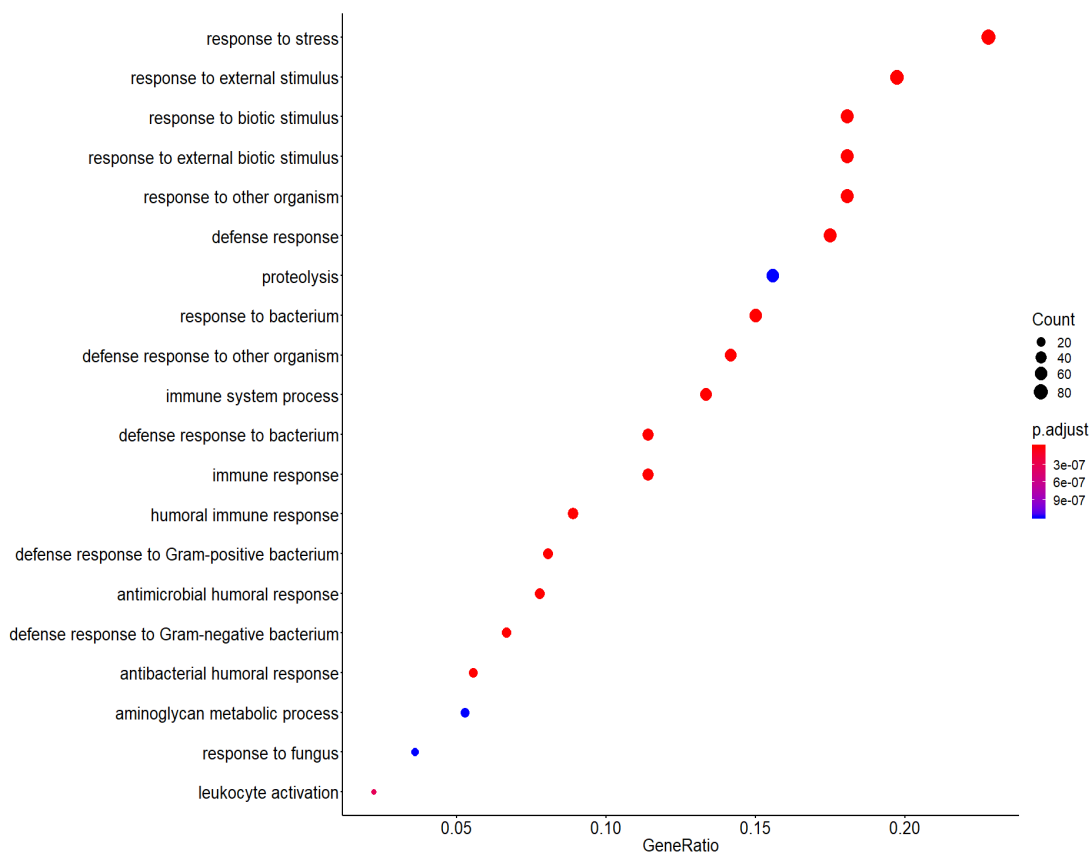

B

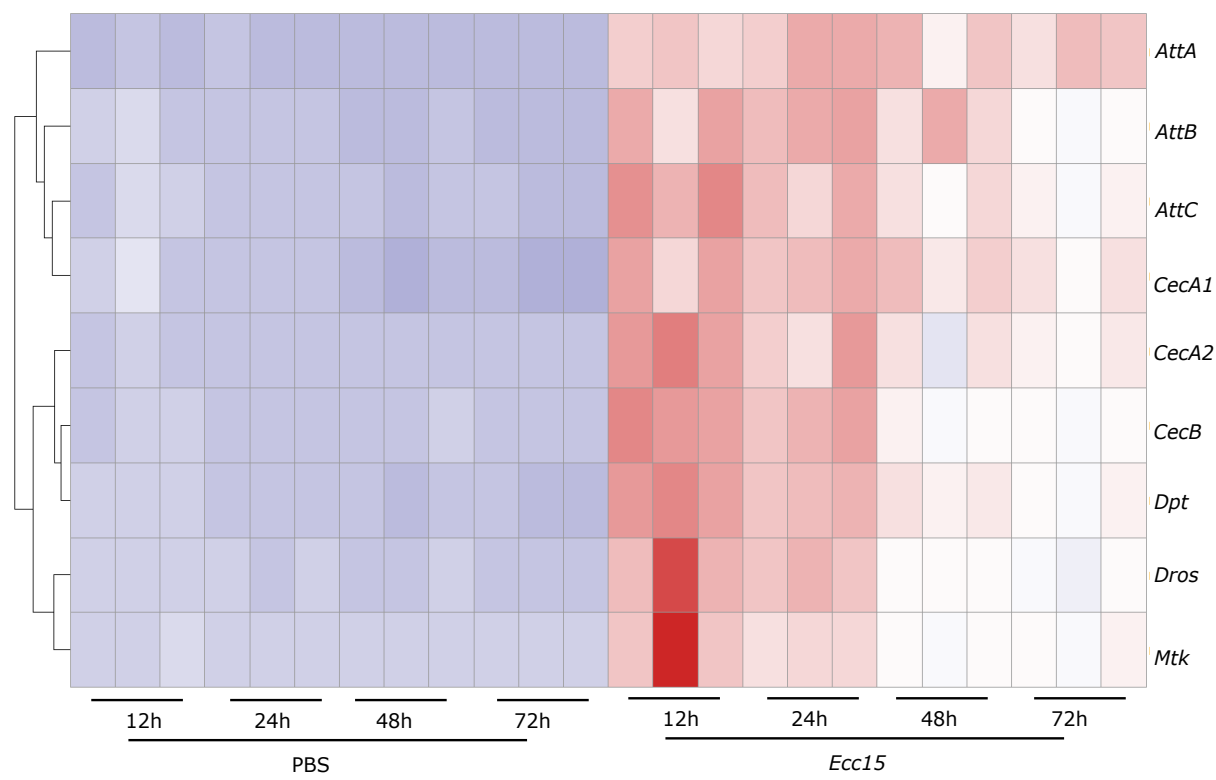

Fig S2

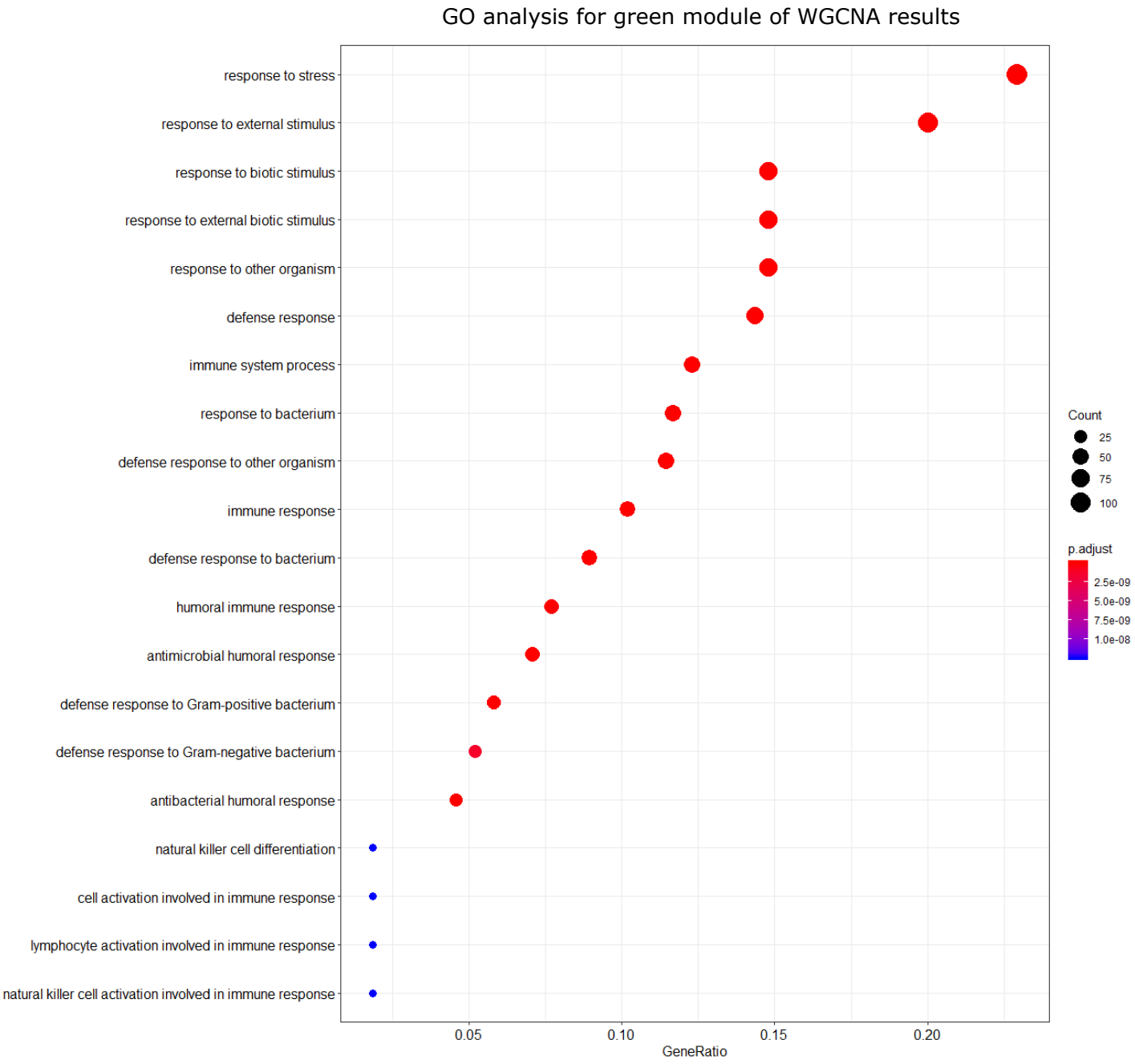

Fig S3

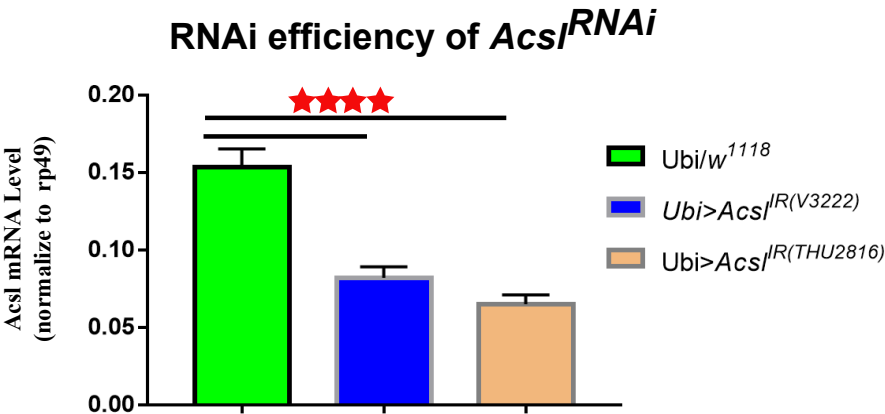

Supplement: Supplementary Figure 1 — GO analysis and Heatmap for up-regulated genes after Ecc15 infection. (A1-A4) GO analysis for the up-regulated genes at indicated time points (12, 24, 48, 72 hpi). (B) Heatmap plot for AMPs genes of Imd pathway at indicated time points (12, 24, 48, 72 hpi). [file Image_1.pdf]
